# Easing the burden: exploring the role of long-acting testosterone formulations in gender-affirming care

**DOI:** 10.3389/fpubh.2026.1751013

**Published:** 2026-02-11

**Authors:** Rahil Hudda, Vi Nguyen, Ravi Iyengar, Allan Pfeil, T. Mike Hsieh, Jill Blumenthal

**Affiliations:** 1 Department of Family Medicine, University of California San Diego Medical Center, San Diego, CA, United States; 2 Department of Urology, University of Washington Medical Center, Seattle, WA, United States; 3 Department of Internal Medicine, University of California San Diego Medical Center, San Diego, CA, United States; 4 Department of Urology, University of California San Diego Medical Center, San Diego, CA, United States

**Keywords:** gender dysphoria, gender-affirming care, hormone replacement therapy, injectable, long-acting, testosterone, transgender, transgender health disparities

## Abstract

**Introduction:**

Long-acting testosterone formulations are essential but infrequently used for gender affirmation. Although not FDA approved in the United States for gender dysphoria, testosterone undecanoate is a long-acting testosterone formulation administered every 10 weeks. Similarly, Testopel is an FDA-approved testosterone pellet implanted subcutaneously every three to six months, which slowly releases testosterone for a long-acting androgenic effect.

**Methods:**

A retrospective review was conducted between January 2020 and June 2024. Data was collected through the electronic health record using a standardized Case Report Form and patients were identified using a unique study identifier corresponding to their medical record number. Data was then collected about these patients including sex assigned at birth, gender identity, age, race, insurance coverage type, prior testosterone formulation use, type of LA testosterone used, duration of LA testosterone use, reasons for LA testosterone use, recent testosterone level, and recent hematocrit level.

**Results:**

Thirteen individuals were found. Median age was 37 years (IQR 23, 53), and 54% were White, 15% Latino/Hispanic, 7.7% Asian, 8% Black, 8% Mixed Race, and 8% American Indian. Insurance coverage included 8% Medicaid, 78% private, and 15% Medicare. Most patients (85%) were on testosterone undecanoate while 15% were on Testopel. Median duration of use was 20 months (IQR 5, 102). Formulations prior to switching were 69% short-acting testosterone injections, 23% topical gel, and 8% patches. Findings showed most common reasons for switching to long-acting testosterone formulations were 31% poor self-reported adherence (31%) and intolerance or needle phobia (31%). Median total testosterone level was found to be 403 (IQR 167–689) and median hematocrit of 47.2% (IQR 27.6–48.3).

**Conclusion:**

Many chose LA testosterone to improve adherence, which ultimately would improve patient satisfaction. Although there are no official guidelines recommending short versus long-acting testosterone use, having the availability of long-acting formulations may create space for shared decision-making between patients and providers to best address gender-affirming goals of patients. Barriers may exist in terms of paying for and acquiring long-acting formulations, making a dedicated pharmacy technician or insurance authorization specialist a critical part of a gender health program.

## Introduction

Gender affirming hormone therapy (GAHT) plays a crucial role in reducing gender dysphoria and improving mental health outcomes among transgender and nonbinary individuals (1, 2). For individuals assigned female at birth, testosterone therapy remains the primary pharmacologic intervention (3).

There are various formulations that exist for testosterone, including transdermal, subcutaneous (SC), intramuscular (IM), oral, and long-acting preparations (3–6). Short-acting injectables (e.g., testosterone cypionate) are commonly used in the United States, often administered weekly or biweekly (3). This schedule may create barriers for some individuals, contributing to poor adherence, fluctuating serum testosterone levels, and injection fatigue.

Testosterone undecanoate is a long-acting intramuscular formulation that was approved by the FDA in 2014 for male hypogonadism (7). Its long-acting effects provide for a more extended dosing interval of approximately 10 weeks (7). Although it is used off label in the United States, it is commonly and widely used internationally for the purposes of gender dysphoria. Use of this formulation in the US requires strict administration guidelines due to concerns for rare reactions including pulmonary oil microembolisms and anaphylaxis (7, 8). Because of these concerns, the Risk Evaluation and Mitigation Strategy (REMS) program was created in the United States, requiring administration of testosterone undecanoate to be done only by a trained healthcare professional followed by post-injection observation for thirty minutes (7–9). Testopel, another long-acting testosterone formulation, is an FDA approved subcutaneous testosterone pellet that delivers sustained androgenic effects for up to six months (10, 11). Recent studies in transgender men report favorable virilizing effects and convenience, but also note procedure-related complications (pellet extrusion and site infection), highlighting the need for routine monitoring (12).

Data on the use of long-acting testosterone formulations for gender affirming care is limited, especially in the United States. This study aims to describe a cohort of transmasculine individuals using long-acting testosterone for gender-affirmation and the reasons for this particular delivery method at two Academic Health Center clinics in Southern California.

## Materials and methods

A retrospective review was conducted at two Academic Health Center clinics offering hormone therapy to transgender and nonbinary patients. The study included individuals who initiated either testosterone undecanoate or Testopel long-acting formulations for gender affirmation between January 2020 (at initial initiation of long-acting testosterone) and June 2024. (at the time of data extraction). The research protocol was approved and deemed exempt by the Institutional Review Board at the University of California, San Diego on April 28th, 2024.

Eligible patients were identified using electronic health records and assigned unique study identifiers. Data was extracted using a standardized case report form with the following inclusion criteria: assigned female at birth, identified as transmasculine or non-binary, and initiated long-acting testosterone for gender affirmation purposes. Data was collected about the patients including: sex assigned at birth, gender identity, age, race, insurance coverage type, prior testosterone formulation, type of LA testosterone, duration of LA testosterone use, reasons for LA testosterone use, recent testosterone level, and recent hematocrit level.

## Results

Thirteen individuals assigned female at birth initiated long-acting testosterone for gender affirmation. Twelve identified as transgender men and one as non-binary. The median age was 37 years (IQR 23, 53). Race and ethnic composition were as follows: 54% White, 15% Latinx/Hispanic, 8% Black, 8% Mixed race, 8% American Indian, and 8% Asian. Most patients (78%) were privately insured, with 15% covered by Medicare and 8% by Medicaid. 85% were on testosterone undecanoate while 15% were taking Testopel. Median duration of long-acting testosterone use was 20 months (IQR 5, 102) (Table 1).

**Table 1:** Characteristics of participants using long-acting testosterone (*N* = 13).

Characteristic	*n* (%) or Median (IQR)
Gender identity	
• Transgender male	12 (92%)
• Nonbinary	1 (8%)
Age (years)	37 (IQR 23, 53)
Race/Ethnicity	
• White	7 (54%)
• Latino/Hispanic	2 (15%)
• Asian	1 (8%)
• Black	1 (8%)
• Mixed race	1 (8%)
• American Indian	1 (8%)
Insurance type	
• Private	10 (78%)
• Medicare	2 (15%)
• Medicaid	1 (8%)
Type of long-acting testosterone	
• Testosterone undecanoate	11 (85%)
• Testopel pellets	2 (15%)
Duration of LA Use (months)	20 (IQR 5, 102).
Testosterone formulations prior to LA	
• Short-acting injectable	9 (69%)
• Topical gel	3 (23%)
• Patch	1 (8%)
Median total testosterone	403 (IQR 167–689)
Median hematocrit	47.2% (IQR 27.6–48.3)

**Figure 1:**
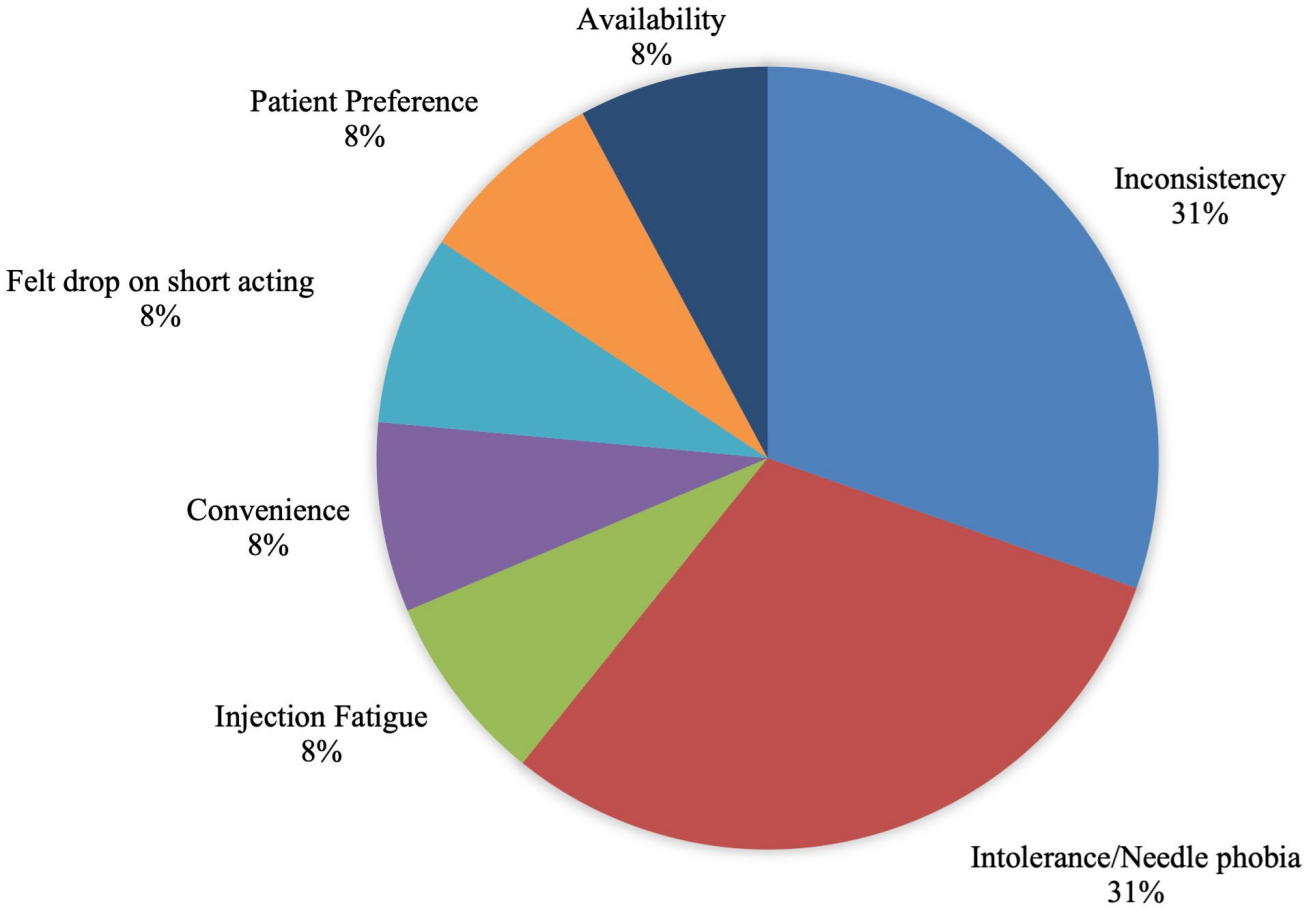
Reasons for long-acting testosterone use.

Formulations prior to switching were 69% short-acting testosterone injections, 23% topical gel, and 8% transdermal patches. Reported reasons for transitioning to long-acting testosterone included poor adherence (31%), intolerance/needle phobia (31%), injection fatigue (8%), convenience (8%), fluctuations on short-acting testosterone (8%), patient preference (8%), and international availability (8%) (Figure 1).

Lab data available prior to the last dose administered demonstrated median total testosterone levels of 403 (IQR 167–689) and median hematocrit of 47.2% (IQR 27.6–48.3), consistent with therapeutic androgenization. No cases of pulmonary oil microembolism or anaphylaxis were observed in this cohort.

## Discussion

This study describes one of the few United States based cohorts of transgender and nonbinary individuals assigned female at birth receiving long-acting testosterone for gender affirmation. Most of the participants transitioned to long-acting formulations from short-acting ones, citing adherence challenges, needle intolerance, and convenience as primary motivators. Gender-affirming hormone therapy is a life-long process, and these findings underscore the importance of flexible, patient-centered treatment modalities.

The pharmacokinetic profile of testosterone undecanoate and Testopel offers stable serum testosterone concentrations at extended dosing intervals, potentially reducing hormonal fluctuations and improving quality of life (7, 10, 11). With respect to testosterone undecanoate, international and pharmaceutical data suggest that pulmonary oil microembolisms and anaphylaxis are rare, occurring in fewer than 0.01% of injections (7, 8, 13). While the REMS program serves as a safety measure for reducing adverse events, the risk for these rare events are so low that it may also serve as a barrier for patients from accessing this formulation. Routine implementation of both testosterone undecanoate and Testopel in clinical settings faces shared challenges related to procedural access, insurance coverage, and variable absorption rates. Nonetheless, it remains a viable alternative for individuals preferring less frequent administration or those who face difficulties with injections.

Erythrocytosis is a known effect of testosterone therapy and a key safety consideration in prescription GAHT. The degree of elevation can vary based on formulation, dosing, and cumulative testosterone exposure. Short-acting formulations, especially administered at longer intervals, have been associated with higher peak serum testosterone levels and higher prevalence of erythrocytosis compared to transdermal formulations (14–16). Long-acting formulations provide more stable serum testosterone levels and may reduce peak-related erythropoietic stimulation; however, erythrocytosis is still reported with long-term use (14, 15). Similarly, testosterone pellets offer sustained androgen delivery but may limit dosing flexibility once implanted, which can complicate management of rising hematocrit. Studies have reassuringly shown that hematocrit levels with these formulations stay within acceptable ranges (11, 17). These findings highlight that the risk of erythrocytosis among formulations are shared and emphasize the importance of routine monitoring.

Financial and logistical barriers remain major impediments to the use of long-acting testosterone formulations (18). Short-acting injectable testosterone is typically the least expensive, with costs out of pocket around $15–$60 per vial (19). These lower costs are largely attributable to the long -standing availability of these formulations, their stability and extended shelf life, and widespread generic manufacturing, which together contribute to more consistent insurance coverage (19). Out-of-pocket costs for testosterone undecanoate vary significantly, but with commercial insurance and the manufacturer’s copay program, eligible patients can pay $0 per injection, though the drug’s retail price can be around $2,000 per shot (7, 19). Testosterone pellets similarly on average cost $1,000 per procedure (19). Cost differences are compounded by variable insurance coverage, prior authorization requirements, and possible out-of-pocket expenses for monitoring and provider visits which can create their own barriers (9, 18).

Integrating dedicated access personnel (e.g., pharmacists, pharmacy technicians, patient navigators) within gender health programs can help streamline processes such as prior authorizations, REMS program requirements, and other logistical steps, ultimately improving access to therapy.

## Limitations

This study has several limitations. Its small sample size and single-institution setting may restrict the generalizability of the findings. The retrospective design further limits causal inference. In addition, long-term outcomes and patient satisfaction data were not systematically collected. As a result, the findings may not be fully representative of all transmasculine individuals or clinical settings. Future prospective multicenter studies are warranted to better characterize rare adverse events, assess patient-reported outcomes, and inform evidence-based guidelines for the use of long-acting testosterone in gender-affirming care in the United States.

## Conclusion

Long-acting testosterone formulations, including testosterone undecanoate and Testopel, represent crucial options in gender-affirming trans masculinization and expand therapeutic options. These formulations offer more consistent androgen exposure and reduced dosing frequency (7, 11). Having access to these formulations offers improved adherence, overall patient satisfaction, and quality of life. Despite regulatory and cost barriers, expanding access to long-acting testosterone formulations supports individualized, affirming, and sustainable hormone therapy that is safe and effective for transgender and nonbinary individuals.

## Data Availability

The original contributions presented in the study are included in the article/supplementary material, further inquiries can be directed to the corresponding author/s.
